# Altered Distribution of SNARE Proteins in Primary Neurons Exposed to Different Alpha-Synuclein Proteoforms

**DOI:** 10.1007/s10571-023-01355-3

**Published:** 2023-05-02

**Authors:** Emma Brolin, Martin Ingelsson, Joakim Bergström, Anna Erlandsson

**Affiliations:** 1 Department of Public Health and Caring Sciences/Molecular Geriatrics, Rudbeck Laboratory, Uppsala University, SE-752 37, Uppsala, Sweden; 2 Krembil Brain Institute, University Health Network, Toronto, ON Canada; 3 Department of Medicine and Tanz Centre for Research in Neurodegenerative Diseases, University of Toronto, Toronto, ON Canada

**Keywords:** Alpha-synuclein, SNARE, Proximity ligation assay, Synapse, Primary neurons

## Abstract

Growing evidence indicates that the pathological alpha-synuclein (α-syn) aggregation in Parkinson’s disease (PD) and dementia with Lewy bodies (DLB) starts at the synapses. Physiologic α-syn is involved in regulating neurotransmitter release by binding to the SNARE complex protein VAMP-2 on synaptic vesicles. However, in which way the SNARE complex formation is affected by α-syn pathology remains unclear. In this study, primary cortical neurons were exposed to either α-syn monomers or preformed fibrils (PFFs) for different time points and the effect on SNARE protein distribution was analyzed with a novel proximity ligation assay (PLA). Short-term exposure to monomers or PFFs for 24 h increased the co-localization of VAMP-2 and syntaxin-1, but reduced the co-localization of SNAP-25 and syntaxin-1, indicating a direct effect of the added α-syn on SNARE protein distribution. Long-term exposure to α-syn PFFs for 7 d reduced VAMP-2 and SNAP-25 co-localization, although there was only a modest induction of ser129 phosphorylated (pS129) α-syn. Similarly, exposure to extracellular vesicles collected from astrocytes treated with α-syn PFFs for 7 d influenced VAMP-2 and SNAP-25 co-localization despite only low levels of pS129 α-syn being formed. Taken together, our results demonstrate that different α-syn proteoforms have the potential to alter the distribution of SNARE proteins at the synapse.

**Graphical Abstract:**

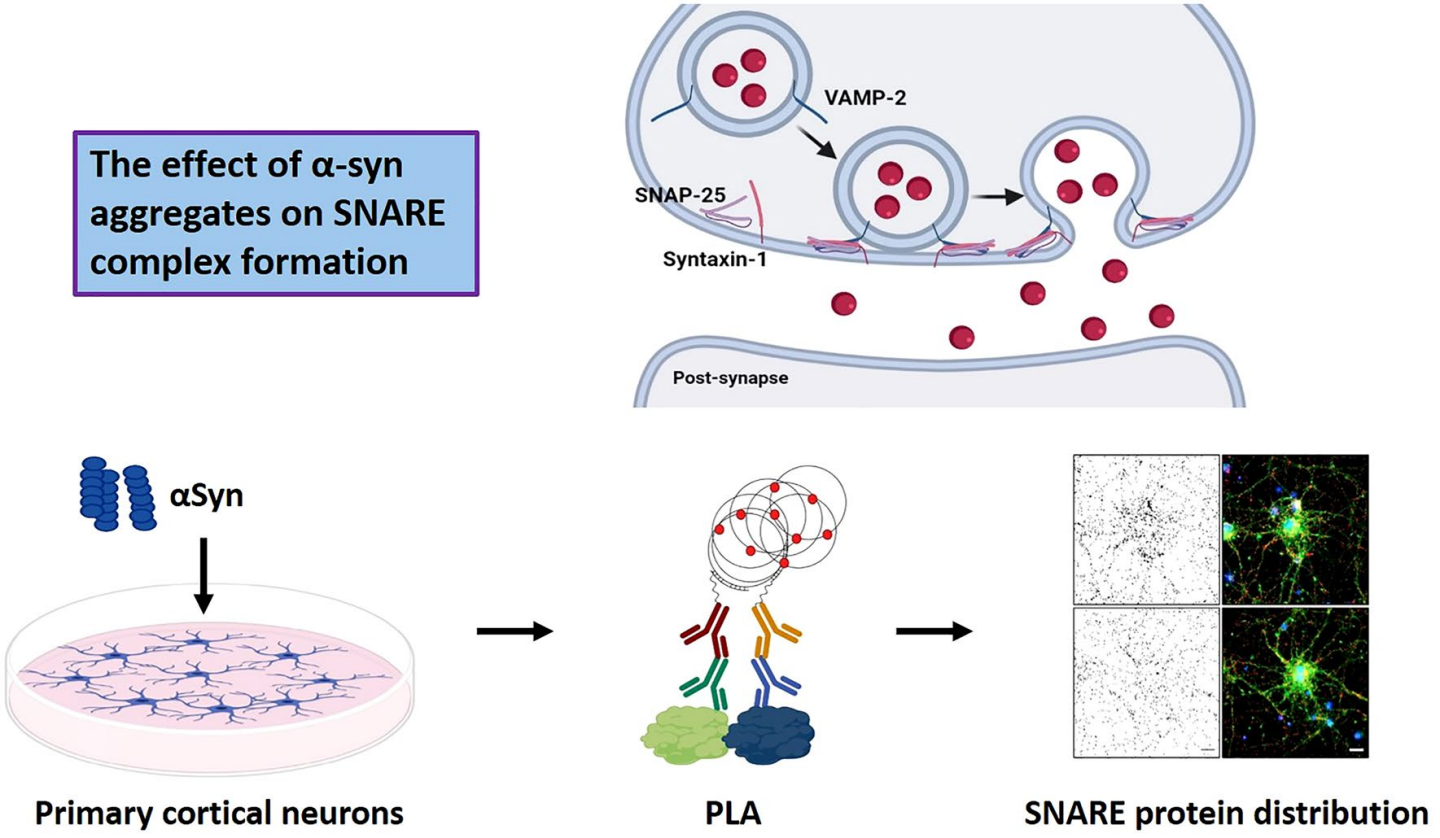

## Introduction

Aggregation of the presynaptic protein alpha-synuclein (α-syn) into filamentous inclusions termed Lewy bodies and Lewy neurites is characteristic for α-synucleinopathies, including Parkinson´s disease (PD) and dementia with Lewy bodies (DLB). These conditions are slowly progressive, with pathology emerging over decades. During disease development, α-syn aggregates are believed to spread between cells and brain areas in a prion-like manner, causing degeneration of synapses and neuronal cell bodies, years before the onset of the clinical symptoms (Braak et al. 2003; Cheng et al. 2010; Goedert 2015).

Alpha-synuclein is abundantly expressed in neurons and is particularly enriched at the presynaptic terminals (Sharma and Burre 2023). The aggregation cascade of α-syn follows a nucleation–elongation pathway, where monomers with a partly folded structure form oligomeric intermediates that aggregate further into fibrils. Increasing evidence suggests that the α-syn pathogenesis starts at the synapses (Sharma and Burre 2023). In the DLB brain, a vast majority (~ 90%) of the aggregated α-syn is located at the presynapse (Kramer and Schulz-Schaeffer 2007). In addition, presynaptic α-syn oligomers are frequently present in transgenic PD mouse models (Tanji et al. 2010; Spinelli et al. 2014). Importantly, endogenous α-syn fibrillation can be initiated and accelerated (i.e., seeded), both in neuronal cell models and in vivo, by the addition of in vitro produced α-syn aggregates.

The complete physiological function of α-syn is still unclear, but compelling data indicate that α-syn is involved in the regulation of neurotransmitter release and synaptic vesicle (SV) clustering and recycling (Yoo et al. 2023; Scott and Roy 2012; Burre et al. 2010). For example, α-syn has been proposed to promote SNARE complex formation by binding to VAMP-2 (Sun et al. 2019). The SNARE complex is vital for fusion of the SV membrane and the plasma membrane during neurotransmitter release. It consists of the vesicle associated SNARE protein, VAMP-2, which binds to the plasma membrane associated SNARE proteins SNAP-25 and syntaxin-1 (Sudhof and Rothman 2009).

In vitro lipid fusion experiments suggest that aggregation of α-syn can affect SNARE complex formation and SV clustering (Hawk et al. 2019; Lai et al. 2014; Choi et al. 2013). Moreover, analyses of transgenic PD mice and non-tg mice with adenovirally overexpressed human α-syn showed altered distribution of the SNARE proteins (Faustini et al. 2018; Garcia-Reitbock et al. 2010). Interestingly, the SV release machinery, including the SNARE proteins, has also been implicated to be involved in propagation of α-syn aggregates (Okuzumi et al. 2018). However, the exact pathophysiological link between α-syn and the SV release machinery remains to be elucidated.

The aim of this study was to investigate both short- and long-term effects of exogenously added α-syn monomers and PFFs on the distribution of SNARE proteins in murine primary neurons. Furthermore, we investigated if extracellular vesicles (EVs) derived from astrocytes treated with α-syn monomers or PFFs could alter SNARE protein distribution.

## Materials and Methods

### Generation and Characterization of α-syn PFFs

Prior to aggregation, recombinant mouse α-syn monomers (RP-009, Proteos, Kalamazoo, MI) were centrifuged at 15 000 × g for 10 min at 4 °C to ensure that no aggregates were present and the concentration was measured by nanodrop (ε for mouse α-syn: 7450 M-1 cm-1, DeNovix). The monomer solution was then diluted to 5 mg/ml with 2 × PBS (Thermo Fischer Scientific and filtered through a 0.45 µm cellulose acetate filter (Costar spin-x) at 16 000 × g for 5 min. Part of the monomer sample was stored at − 70 °C. The rest of the sample was vortexed at high speed for 3 s and placed on a shaker for 7 d at 37 °C for generation of PFFs that were then stored at − 70 °C until use. Prior to the addition to cells, the PFFs were diluted to 2 mg/ml and sonicated (Sonics) for 1 min with 1 s on, 1 s off cycle at 20% amplitude on ice. The monomer sample was not sonicated.

### Transmission Electron Microscopy

A 5 µl drop of the α-syn sample was placed on a formvar and carbon coated 200-mesh copper grid. The excess solution was removed by blotting with filter paper. The sample was then directly contrasted with 2% uranyl acetate. Excess of uranyl acetate was removed by blotting on filter paper. Dried grids were examined by Tecnai™ G2 Spirit BioTwin transmission electron microscope (Thermo Fisher/FEI) at 80 kV with an ORIUS SC200 CCD camera and Gatan Digital Micrograph software (both from Gatan Inc.).

### Animals

All animal experiments were approved by the Uppsala County Animal Ethics board (5.8.18–08,472/18) following the rules and regulations of the Swedish Animal Welfare Agency and in compliance with the European Communities Council Directive of 22 September 2010 (2010/63/EU). The mice were housed in a 12 h dark–light cycle, in an enriched environment and had access to food and water ad libitum.

### Primary Neuron Cultures

Cerebral cortices were dissected from C57/BL6 mice embryos (E14) in Hank’s buffered salt solution (HBSS) supplemented with 50 U/ml Penicillin, 50 mg/ml Streptomycin, and 8 mM Hepes buffer (ThermoFisher Scientific). The cortices were centrifuged in fresh HBSS for 3 min at 150 × *g* and then resuspended and dissociated into a homogenous solution. Any remaining blood vessels were allowed to sediment for 10 min. The supernatant was transferred to a new tube and centrifuged for 5 min at 150 × *g*. The cell pellet was carefully resuspended in neurobasal medium supplemented with B27, 100 U/ml penicillin, 100 μg/ml streptomycin, and L-glutamine 2 mM (Thermo Fisher Scientific) and plated at 90 000 cells/ml on poly-L-ornithine (Sigma-Aldrich) and laminin (Thermo Fisher Scientific)-coated cover slips.

### Short-term α-syn Exposure

Mouse α-syn monomers, PFFs (140 nM), or PBS was added to neurons at 19 or 20 days in vitro (DIV). The cultures were fixed in 4% paraformaldehyde (PFA) after 2-h or 24-h incubation with α-syn (at 20 DIV).

### Long-term α-syn Exposure

Mouse α-syn monomers (70 nM), PFFs (70 nM), or PBS was added to neurons at 12 DIV. The neurons were then cultured for an additional 7 d. During this period, half of the culture medium was changed every 2–3 d (without adding any α-syn). The cultures were fixed in 4% paraformaldehyde (PFA) at 19 DIV.

### Long-term α-syn Exposure in Presence of Chariot™

Chariot™ protein delivery reagent (Active Motif) was used to enhance the PFF uptake. Alpha-synuclein monomers, PFFs, or PBS was mixed with Chariot™ for 30 min prior to them being added to the neurons. The Chariot™-α-syn mixture was added dropwise followed by the addition of fresh culture medium (final concentration 70 nM for both monomer and PFFs). After 1-h incubation at 37 °C, 1 ml of medium was added and the cells were cultured for 7 d. During this period, half of the culture medium was changed every 2–3 d (without adding any α-syn or Chariot™). The cultures were fixed in 4% paraformaldehyde (PFA) at 19 DIV.

### Astrocyte Cultures and Extraction of Extracellular Vesicles

E14 mouse cerebral cortexes were dissected and dissociated in HBSS supplemented with 100 U/ml penicillin, 100 μg/ml streptomycin, and 8 mM HEPES buffer. The embryonic cortical stem cells were allowed to expand as neurospheres in DMEM/F12 GlutaMax medium (Invitrogen) supplemented with B27 (Invitrogen), 100 U/ml penicillin, 100 μg/ml streptomycin, 8 mM HEPES buffer, 10 ng/ml bFGF (Invitrogen, diluted in 10 mM Tris–HCl (pH 7.6) + 0.1% BSA and PBS), and 20 ng/ml EGF (BD biosciences), dissolved in MQ water). For experiments, the neural stem cells were seeded in a monolayer at a density of 3 × 104 cells/cm^2^ on cover glasses coated with poly-L-ornithine and laminin and differentiated to astrocytes in DMEM/F12 medium with GlutaMAX, 1 × B27 supplement, 100 U/ml Penicillin, 100 µg/ml Streptomycin, 8 mM Hepes buffer, and human CNTF recombinant protein (10 ng/ml, Gibco). The medium was fully replaced every 2–3 d during the seven-day differentiation period. Alpha-synuclein (monomers or PFFs, 500 nM) was added to astrocytes at 7 d of differentiation. After 24-h incubation with α-syn, the astrocytes were washed 3 times with medium and cultured for an additional 12 d in α-syn-free medium. Conditioned medium was collected at days 6 and 12 and pooled. Before ultracentrifugation, larger debris was removed by centrifugation at 300 xg for 5 min followed by and additional centrifugation at 2000 xg for 10 min. The supernatant was ultracentrifuged in a S50A rotor at 135 000 xg for 1.5 h. The EV containing pellet was resuspended in neurobasal medium supplemented with B27, 100 U/ml penicillin, 100 μg/ml streptomycin, and 2 mM L-glutamine and added to the neurons at 12 DIV. The neuronal cultures were fixed in 4% PFA at 19 DIV.

### Immunocytochemistry

The fixed neurons were permeabilized and blocked in 0.1% Triton X-100 and 5% normal goat serum (NGS) in PBS for 30 min at room temperature (RT). Primary antibodies were diluted in PBS containing 0.5% NGS and added to the cultures for 1–2 h at RT. The primary antibodies used were as follows: rabbit monoclonal ab51253 anti-pS129 α-syn (Abcam, 4 µg/ml), mouse monoclonal TuJ-1 anti-βIII-tubulin (BioLegend, 1:200), and mouse monoclonal 110 111 (clone 78.3) anti-syntaxin-1A (Synaptic systems, 1:250). After washing in PBS, the cells were incubated with Alexa fluor secondary antibodies goat anti-rabbit 594, 1:1000 and goat anti-mouse 488, 1:200 (Life Technologies) for 1 h at RT. The cells were washed in PBS and mounted using Everbright hard set mounting medium with DAPI (Biotium).

### Proximity Ligation Assay

The proximity ligation assay (PLA) was performed on the fixed primary neurons using the Duolink PLA technology probes and reagents (Sigma-Aldrich) as previously described (Almandoz-Gil et al. 2018). Briefly, the cells were permeabilized with 0.4% Triton X-100 in PBS for 10 min at RT, washed with PBS, and then blocked with Duolink blocking solution for 30 min at 37 °C. The primary antibodies were diluted in Duolink antibody diluent and added to the cells for 1–2 h at RT. The primary antibodies used were as follows: rabbit monoclonal anti-VAMP-2 EPR12790 (Abcam, 1:250), mouse monoclonal anti-syntaxin-1A 110,111 (Synaptic Systems, 1:250), rabbit monoclonal anti-SNAP-25 EP3274 (Abcam, 4 µg/ml), and mouse monoclonal anti-SNAP-25 SP12 (Santa Cruz, 1:50). The cultures were washed with Duolink wash buffer A prior to incubation with the PLA probes PLUS and MINUS (diluted in antibody diluent) for 1 h at 37 °C. The cells were then washed with wash buffer A and incubated for 30 min at 37 °C in ligation stock buffer containing ligase. Then, the cells were washed in wash buffer A and incubated in the amplification solution with polymerase and the fluorophore-labeled oligonucleotides for 100 min at 37 °C. After washing with Duolink wash buffer B, the actin was stained using Phalloidin-FITC (Sigma- Aldrich) at 1.25 µg/ml for 20 min. Finally, the cells were washed with PBS, followed by washing in 0.01 × wash buffer B and mounted using Duolink in situ mounting medium with DAPI.

### Microscopy and Image Analysis

The stained cells were imaged using a Zeiss Axio Observer Z1 (Zeiss, Oberkochen, Germany) microscope. For quantification, each experiment was performed in triplicates and 10 randomized pictures were captured from each replicate, except for the astrocyte seeding experiment for which 5 randomized pictures were captured per replicate. The microscope settings were kept constant for all images in each experiment to enable direct comparisons between the groups. The proximity ligation assay puncta were quantified using ImageJ by first subtracting the background with the sliding paraboloid. The threshold was set constant and the PLA puncta were separated using the watershed function before the number of PLA puncta was quantified with the analyze particles tool. The number of PLA puncta was normalized to the area of phalloidin.

### Statistical Analysis

GraphPad Prism was used for analysis of data and the generation of graphs. The raw data were analyzed for normal distribution with D’Agostino and Pearson omnibus normality test. Non-parametric tests were applied when data did not fulfill normality criteria. Kruskal–Wallis test with Dunn’s multiple comparison post hoc test was used for analysis of short-term treatment experiments and cells treated with α-syn and Chariot™. The Mann Whitney test was used for analysis of the long-term treatment experiment and the astrocyte EV experiment. Results are presented as mean ± SD and the levels of significance are * *P* < 0.05, ** *P* < 0.01 and *** *P* < 0.001.

## Results

### Generation of Mouse α-syn Preformed Fibrils

**Fig. 1:**
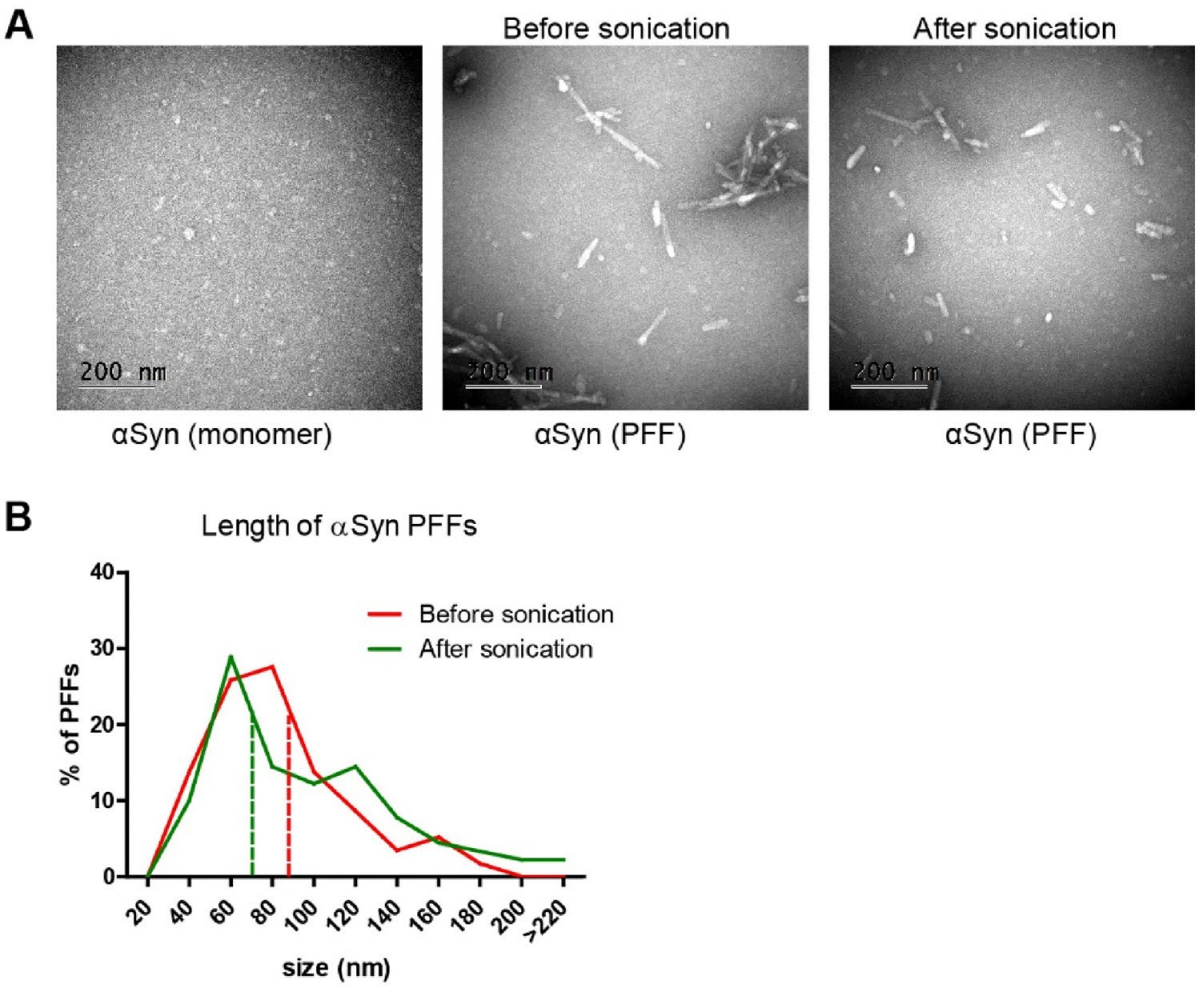
Generation and characterization of α-syn PFFs. **A** Mouse α-syn monomers and PFFs before and after sonication imaged by electron microscopy. Scale bar = 200 nm. **B** Percent of PFFs with a given length before and after sonication. Analysis of 5 images per group. The dashed lines represent the mean length

Recombinant monomeric mouse α-syn was used to generate PFFs, which were further sonicated to facilitate an increased uptake in the cell experiments. Electron microscopy analysis showed that the PFFs were approximately 16 nm wide and 88.1 nm (SD = 50.3 nm) long before sonication, whereas the mean length was reduced to 72.2 nm (SD = 34.0 nm) after sonication (Fig. 1A, B).

### Short-term PFF Exposure Causes Altered SNARE Protein Distribution in Primary Cortical Neurons

**Fig. 2:**
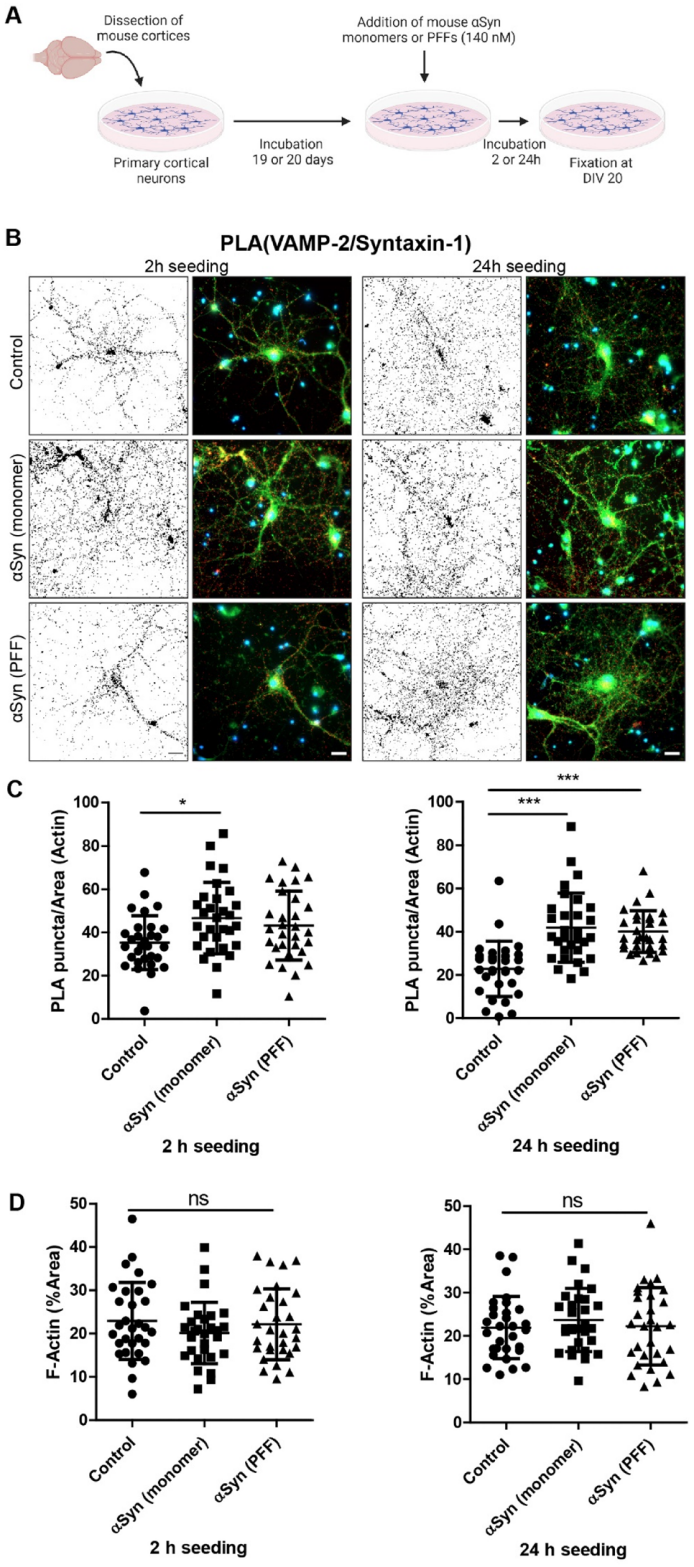

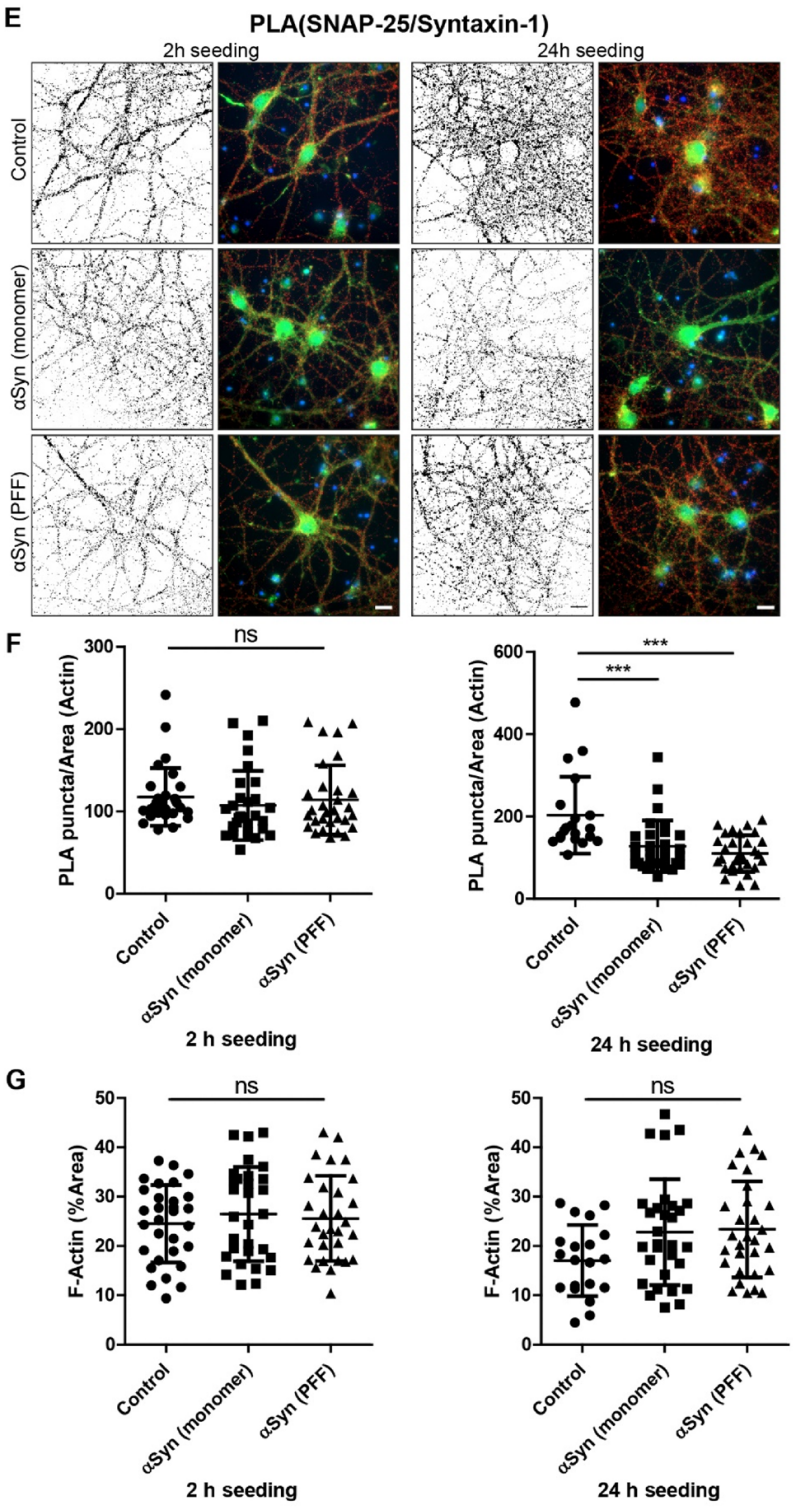
Altered SNARE protein distribution in primary cortical neurons after 24 h PFF exposure. **A** Schematic outline of experimental design. Short-term effects of α-syn monomers or PFFs (140 nM, 2 h or 24 h exposure) on primary neurons were analyzed at 20 DIV. **B** Representative images of PLA between VAMP-2 and syntaxin-1. **C** Quantification of PLA puncta per area of F-actin. **D** Quantification of F-actin after 2- or 24-h incubation. **E** Representative images of PLA between SNAP-25 and syntaxin-1. **F** Quantification of PLA puncta per area of F-actin. **G** Quantification of F-actin after 2- or 24-h incubation. Scale bars = 20 µm. F-actin (green) and DAPI (blue). The black-and-white image depicts the PLA puncta in red in the RGB image. Each experiment was repeated three times and 10 randomly captured images were analyzed per experiment, using Kruskal–Wallis test with Dunn’s multiple comparisons post hoc test (non-significant (ns), * *P* < 0.05, *** *P* < 0.001), data are presented as mean ± SD

Proximity ligation assay is a powerful tool that can detect the co-localization of proteins with high specificity and sensitivity, as a signal is only obtained if the two proteins of interest are within 40 nm of each other (Soderberg et al. 2006). In an attempt to understand how α-syn pathology affect the synaptic vesicle release machinery, we investigated the short-term effect of the α-syn on SNARE protein distribution in primary neurons using PLA (Fig. 2A). We detected only a modest increase of VAMP-2/syntaxin-1 PLA signals in neurons treated with monomers for 2 h (Fig. 2B, C, left). However, neurons treated with either monomers or PFFs for 24 h (Fig. 2B, C, right), showed a robust increase of VAMP-2/syntaxin-1 PLA signals, compared to PBS-treated neurons. The F-actin levels were similar for all three treatment groups (Fig. 2D), indicating that the overall neuronal structure was unaffected.

Next, we performed a PLA against the SNARE proteins SNAP-25 and syntaxin-1 (Fig. 2E), which are not believed to interact with α-syn to the same extent as VAMP-2. No changes in SNAP-25/syntaxin-1 PLA signals were detected in cells treated for 2 h with monomers or PFFs (Fig. 2F, left). However, neurons treated with monomers or PFFs for 24 h, had lower SNAP-25/syntaxin-1 PLA signals compared to the PBS control (Fig. 2F, right). No differences in F-actin levels were observed for the three treatment groups (Fig. 2G).

### Long-term PFF Exposure Result in Reduced VAMP-2 and SNAP-25 Co-localization, Although Modest Induction of ser129 α-syn

We next sought to investigate how the distribution of SNARE proteins was affected in long-term seeding experiments. Based on previously published data, we expected the exogenously added α-syn seeds to initiate aggregation of endogenous α-syn and induce phosphorylation of α-syn at ser129 (pS129) (Patterson et al. 2019; Volpicelli-Daley et al. 2014, 2011). Hence, under long-term conditions, effects on SNARE proteins could potentially be attributed to both the exogenously added α-syn species and the endogenously formed α-syn aggregates.

**Fig. 3:**
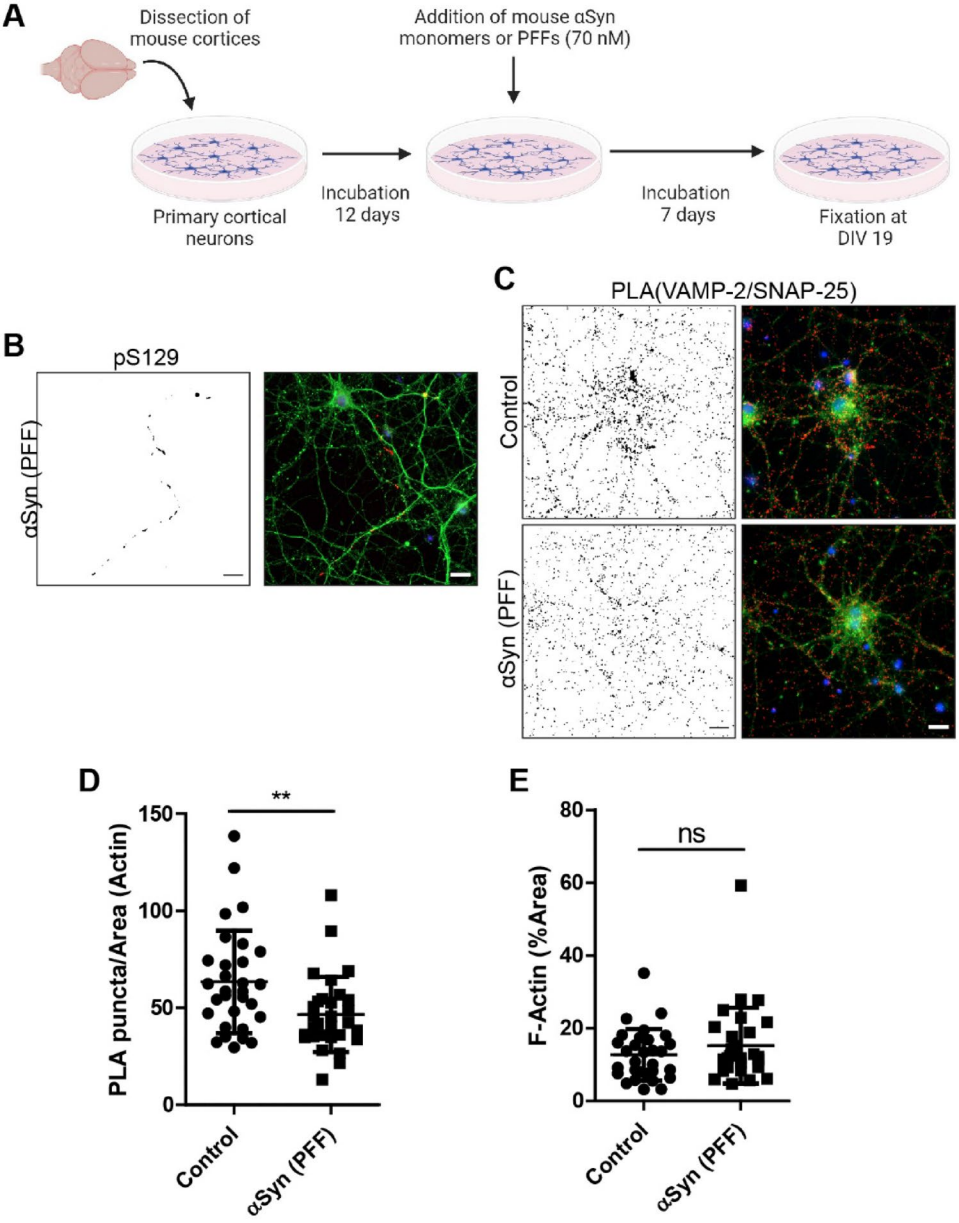
Reduced co-localization of VAMP-2 and SNAP-25 following long-term PFF seeding, despite modest phosphorylation of α-syn. **A** Schematic outline of experimental design. Long-term effects of α-syn monomers or PFFs (70 nM, 7 d of exposure) on primary neurons was analyzed at 19 DIV. **B** Representative image of pS129 α-syn levels in neuronal cultures treated with PFFs. **C** Representative images of PLA between VAMP-2 and SNAP-25 **D** Quantification of PLA puncta per area of F-actin. **E** Quantification of F-actin. Scale bars = 20 µm. B: BIII tubulin (green) and DAPI (blue). C: F-actin (green) and DAPI (blue). The black-and-white image depicts the PLA puncta in red in the RGB image. Each experiment was repeated three times and 10 randomly captured images were analyzed per experiment using Mann Whitney’s test (non-significant (ns), ** *P* < 0.01, *** *P* < 0.001), data are presented as mean ± SD

In the first long-term experimental setup, neurons were exposed to mouse α-syn PFFs at 12 DIV and then cultured for additionally 7 d prior to analysis (Fig. 3A). However, only low pS129 α-syn levels could be detected in the PFF-exposed neurons (Fig. 3B). Nevertheless, cells treated with PFFs had significantly lower VAMP-2/SNAP-25 PLA signals, compared to control cells (Fig. 3C, D). Similar F-actin levels were observed in both cultures (Fig. 3E).

**Fig. 4:**
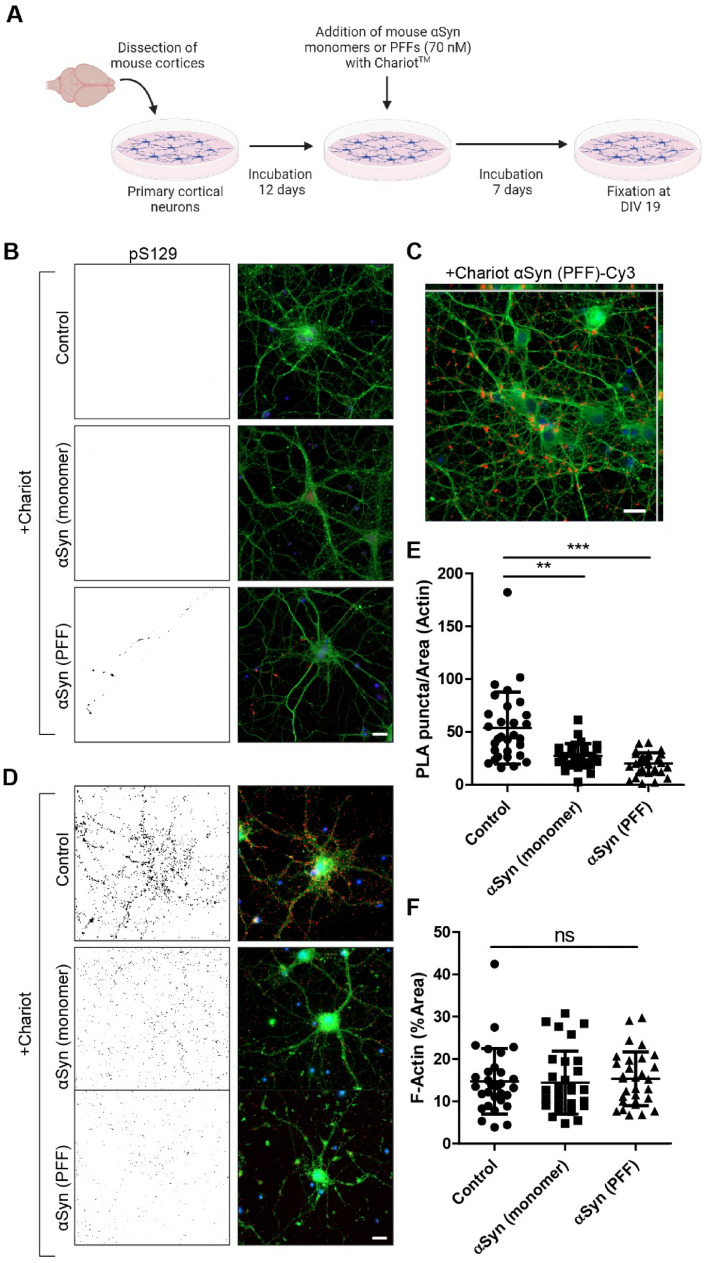
Reduced co-localization of VAMP-2 and SNAP-25 in neurons treated with a combination of Chariot™ and α-syn. **A** Schematic outline of experimental design. Long-term effects of α-syn monomers or PFFs (70 nM, 7 d of exposure with protein delivery reagent Chariot™) on primary neurons was analyzed at 19 DIV. **B** Representative image of pS129 α-syn levels in neuronal cultures treated with α-syn monomers or PFFs in the presence of Chariot™. **C** Representative orthogonal view from a z-stack of uptake of Cy3-labeled α-syn PFFs (red). **D** Representative images of PLA between VAMP-2 and SNAP-25. **E** Quantification of PLA puncta per area of F-actin. **F** Quantification of F-Actin. Scale bars = 20 µm. B and C: BIII tubulin (green) and DAPI (blue). D: F-actin (green) and DAPI (blue). The black-and-white image depicts the PLA puncta in red in the RGB image. Each experiment was repeated three times and 10 randomly captured images were analyzed per experiment using Kruskal Wallis test with Dunn’s multiple comparisons (non-significant (ns), ** *P* < 0.01, *** *P* < 0.001), data are presented as mean ± SD

To further enhance cellular uptake of α-syn seeds, we next added the protein delivery reagent Chariot™ together with the monomers and PFFs at 12 DIV (Fig. 4A). Similar to neurons treated with PFFs alone, the addition of PFFs + Chariot™ only had a modest effect on pS129 α-syn levels (Fig. 4B). Treatment with Chariot™ together with either monomers or PBS did not induce any detectable pS129 α-syn pathology (Fig. 4C). Neurons treated with Cy3-labeled mouse α-syn PFFs and Chariot™ showed a substantial intracellular Cy3-signal at 19 DIV (Fig. 4D), confirming that the seed is indeed being taken up and accumulated. The PFFs were mostly found to be clustered at the neuronal processes, but a subset of PFFs also had a perinuclear localization. Interestingly, a reduced PLA signal between VAMP-2 and SNAP-25 was observed in both neurons treated with Chariot™ + PFFs and Chariot™ + monomers (Fig. 4E). The F-actin levels were unaffected by the addition of monomers or PFFs together with Chariot™ (Fig. 4F).

### Extracellular Vesicles Isolated from Astrocytes with α-syn Pathology Causes co-Localization of VAMP-2 and SNAP-25 in Primary Neurons

**Fig. 5:**
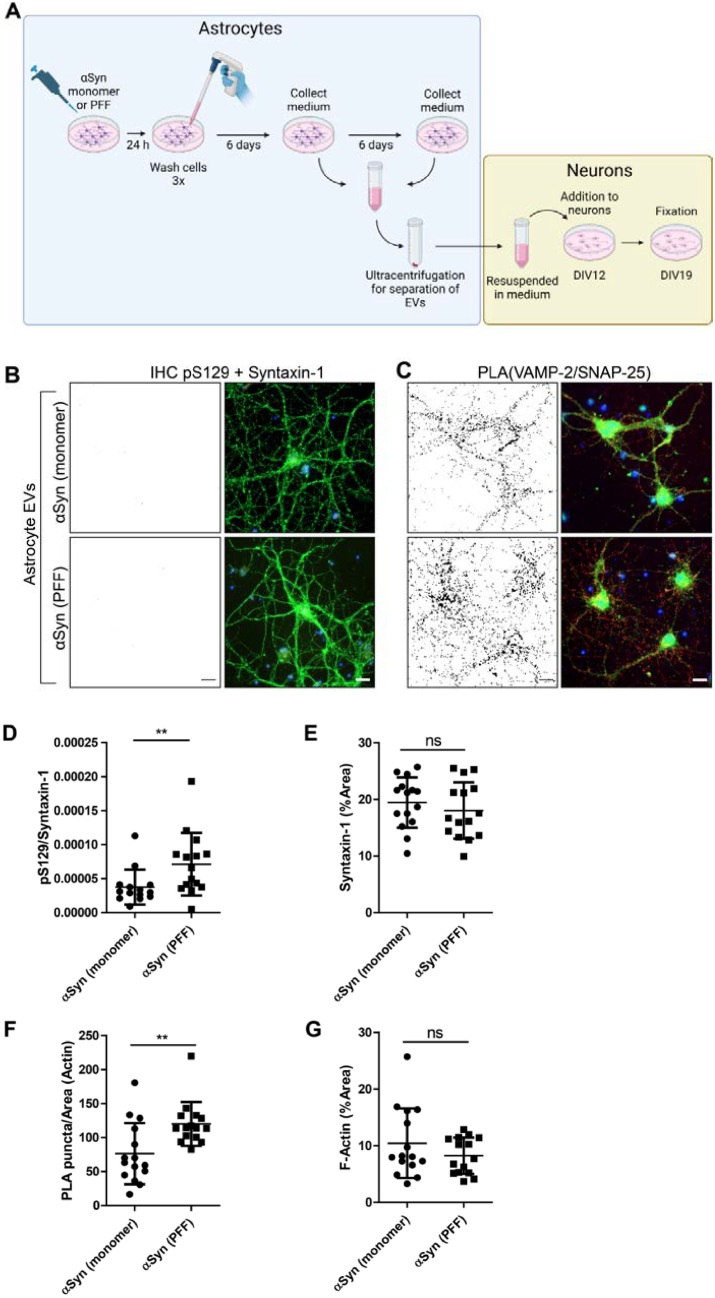
Increased co-localization of VAMP-2 and SNAP-25 in primary neurons exposed to EVs isolated from astrocytes with α-syn pathology. **A** Schematic figure of experimental setup. **B** Representative images of pS129 α-syn in neuronal cultures exposed to EVs from astrocytes that had ingested α-syn monomers or PFFs. The black-and-white image is an inversion of the pS129 α-syn in red in the RGB image to the right. Syntaxin-1 (green) and DAPI (blue). **C** Representative images of VAMP-2/SNAP-25 PLA. **D** Quantification of pS129 α-syn relative to syntaxin-1. **E** Quantification of syntaxin-1. **F** Quantification of PLA puncta relative to area of F-actin. **G** Quantification of F-actin. Scale bars = 20 µm. F-actin stained by phalloidin-FITC (green) and DAPI (blue). The black-and-white image depicts the PLA puncta in red in the RGB image. Each experiment was repeated three times and 5 randomly captured images were analyzed per experiment using Mann Whitney’s test (non-significant (ns), ** *P* < 0.01), data are presented as mean ± SD

We have previously shown that astrocytes engulf and accumulate aggregated α-syn, resulting in severe cellular stress and cell-to-cell transmission of such aggregates, both via tunneling nanotubes and extracellular vesicles (EVs) (Rostami et al. 2021, 2017; Lindstrom et al. 2017). In contrast, monomeric α-syn is immediately degraded by the astrocytes following uptake. Hence, we sought to investigate if EVs released by astrocytes could seed α-syn pathology and/or alter the distribution of SNARE proteins in primary neurons (Fig. 5A). Monomers or PFFs (500 nM) were added to astrocytes for 24 h, after which the α-syn containing medium was removed, and the astrocytes were cultured for additionally 12 d without α-syn. Conditioned medium, collected after 6 and 12 d, was pooled and subjected to ultracentrifugation to collect EVs. The isolated EVs were resuspended and added to neurons at 12 DIV, and the neurons were analyzed 7 d later, at 19 DIV (Fig. 5B-D). The EVs collected from PFF-exposed astrocytes induced a slight increase in pS129 α-syn levels in the neurons, compared to neurons exposed to EVs collected from monomer-exposed astrocytes (Fig. 5B, D). The levels of syntaxin-1 (i.e., number of processes) did not differ between the neurons exposed to EVs from PFF or monomer-astrocytes (Fig. 5E). However, the neurons that received EVs from astrocytes treated with PFFs showed higher VAMP-2/SNAP-25 PLA signals, compared to EVs from monomer-treated astrocytes (Fig. 5C, F). The F-actin levels were similar between the two EV treatment groups (Fig. 5G).

## Discussion

Alpha-synucleinopathies**,** including PD and DLB, are progressive neurodegenerative disorders with a complex pathophysiology that develops over decades. Notably, increasing evidence suggests that the initiation and propagation of α-syn aggregates occur at the presynapse.

The physiological function of α-syn is tightly linked to the presynaptic terminals, where it exerts a dual effect on the synaptic vesicle release machinery. First, it facilitates the formation of the SNARE complex, which is essential to neurons as it regulates the fusion of synaptic vesicles to the plasma membrane, leading to neurotransmitter release (Sudhof 2013). Second, it regulates neurotransmitter release by restricting synaptic vesicle mobilization and recycling (Faustini et al. 2022). However, the interplay between the synaptic vesicle release machinery and pathological α-syn remains poorly defined.

The aim of this study was to investigate changes in the SNARE protein distribution in primary cortical neurons following both short- and long-time exposure to α-syn monomers or PFFs. Both monomeric and aggregated α-syn are known to be endocytosed by primary neurons (Karpowicz et al. 2017; Luk et al. 2009). Moreover, it has been shown that the first sign of α-syn phosphorylation appear at the presynapse in mice injected with PFFs, suggesting that this is the location of uptake (Awa et al. 2022).

We have previously used PLA to visualize co-localization of α-syn and SNARE proteins in murine primary cortical neurons (Almandoz-Gil et al. 2018). In addition, in situ PLA between α-syn and VAMP-2 has been used to detect protein redistribution in mice following AAV injection of human α-syn (Faustini et al. 2018). Here, we performed PLA to measure the co-localization between individual SNARE proteins in murine primary cortical neurons.

First, we treated neurons with α-syn for 2 or 24 h. At these time points, the impact on the SNARE proteins is only due to the exogenous α-syn, as no or very little seeded α-syn pathology has formed at this stage. Notably, treatment with monomers or PFFs affected the distribution of the SNARE proteins without an apparent effect on the overall neuronal viability or structure, as determined by measuring F-actin levels.

Fusion of synaptic vesicles is restricted to a specialized area of the presynaptic plasma membrane, called the active zone (Verhage and Sorensen 2008; Sudhof 2013; Gundelfinger and Fejtova 2012; Zhai et al. 2001). The observed changes on SNARE protein distribution in this study depended on which pair of SNARE proteins targeted. An increased VAMP-2/Syntaxin-1 co-localization was observed after 24 h of treatment with both monomers and PFFs, and already after 2 h with monomers, compared to PBS-treated controls. These results suggest that short-term α-syn treatment caused synaptic vesicles to come in close proximity with the presynaptic active zone. However, a reduced co-localization between SNAP-25/Syntaxin-1 was observed after 24 h of treatment with both monomers and PFFs, indicating an attenuated formation of SNARE complexes. The increased co-localization between VAMP-2 and syntaxin-1 could be attributed the interaction between exogenous α-syn and VAMP-2, whereas SNAP-25 and syntaxin-1 are less known to interact with α-syn (Burre et al. 2014, 2010; Sun et al. 2019).

Previous studies using primary hippocampal neurons have demonstrated that exogenously added α-syn PFFs can initiate aggregation of endogenous α-syn (Volpicelli-Daley et al. 2011; Luk et al. 2009). The endogenous α-syn, but not the exogenously added PFFs, becomes phosphorylated at ser129 and displays a Lewy body-like appearance (Luk et al. 2009). In the second set of experiments, we sought to perform long-term seeding studies by adding PFFs for 7 d to the primary neurons. However, we only detected low levels of pS129 α-syn. Earlier studies have shown that the optimal length of PFFs in order to induce robust pS129 α-syn pathology is less than 50 nm (Patterson et al. 2019). The mean length of the sonicated PFFs used in this study was 72.2 nm, with only 25% of the PFFs being smaller than 50 nm. This could potentially explain the low seeding efficiency compared to previous studies.

In order to increase the pS129 α-syn pathology, we next included the protein delivery reagent Chariot™, which has been shown to facilitate uptake of α-syn aggregates in different cell types, including primary neurons (Fauvet et al. 2012; Bayir et al. 2009; Lee et al. 2009; Larson et al. 2017). The presence of Chariot™ resulted in a robust PFF uptake, but we only detected a slight increase in pS129 α-syn. Despite the fact that PFFs, with or without Chariot™, only induced modest levels of pS129 α-syn pathology, the treatment resulted in a reduced co-localization between VAMP-2 and SNAP-25, supporting that the synaptic vesicles are located away from the active zone. Hence, the effect of long-term exposure to PFF was opposite to what was observed at 24 h. Interestingly, a similar effect was obtained when monomers were added together with Chariot™. Earlier studies have shown that monomeric α-syn is rapidly endocytosed and transferred to lysosomes (Karpowicz et al. 2017). Hence, we expected the monomers to be degraded prior to PLA analysis. However, our data clearly show a reduction in the co-localization of VAMP-2 and SNAP-25 in the presence of monomers + Chariot™. An explanation for this result could be that Chariot™ facilitated uptake of a sufficient amount of monomers to inhibit vesicle release. It is also possible that the degradation of the monomers was affected in the presence Chariot™. In the case of PFF treatment, the effect may instead be explained by the formation of α-syn aggregates, as there was an increase of p129 α-syn levels in these cultures. Importantly, the overall structure of the neurons was unchanged in cells treated with PFFs and/or Chariot™.

Finally, we aimed to investigate if α-syn species secreted from astrocytes could affect the distribution of SNARE proteins in neurons. Astrocytes are crucial for maintaining brain homeostasis by providing physical, energetic, metabolic, and trophic support to neurons and other cell types (Vasile et al. 2017). Hence, functionally impaired astrocytes can be devastating for the brain health in general and neuronal activity in particular. Although α-syn deposits are primarily found in neurons, they also appear frequently in astrocytes at all disease stages in PD/DLB (Braak et al. 2007; Wakabayashi et al. 2000; Terada et al. 2003; Croisier and Graeber 2006; Tu et al. 1998). However, the role of astrocytic α-syn inclusions in the initiation, progression and spread of PD/DLB pathology remains elusive. We have previously shown that astrocytes effectively engulf, but only partially digest α-syn aggregates (Rostami et al. 2021, 2017; Lindstrom et al. 2017). Instead, the pathogenic aggregates are spread to nearby cells via different mechanisms. For example, stressed astrocytes transfer protein aggregates to neighboring cells via thin protrusions called tunneling nanotubes (TNTs) (Rostami et al. 2021, 2017, 2020). Moreover, our previous data demonstrate that incomplete degradation of Aβ in astrocytes results in the secretion of truncated, potentially toxic Aβ peptides (Nikitidou et al. 2017; Sollvander et al. 2016; Beretta et al. 2020). Here, we show that EVs isolated from PFF-exposed astrocytes generated significantly higher levels of pS129 α-syn than EVs from monomer-exposed astrocytes. Importantly, we also observed an increase in VAMP-2/SNAP-25 co-localization in these neurons, indicating that the synaptic vesicles were proximal to presynaptic membranes. However, the effect of EVs from PFF-exposed astrocytes on VAMP-2/SNAP-25 PLA was different to that observed following Chariot™ + PFF treatment. This indicates that the impact of EV-PFF on SNARE homeostasis differs from that of a high concentration of extracellular PFF. We have recently demonstrated that inclusions of aggregated proteins affect the reactive state of astrocytes, as well as their ability to support neuronal function, indicating that stressed astrocytes may contribute to synaptic dysfunction (Konstantinidis et al. 2023b, 2023a).

Taken together, our results show that different α-syn proteoforms can alter the presynaptic distribution of SNARE proteins. However, further studies are necessary to determine exactly how these changes affect the disease pathogenesis of PD/DLB.

## Data Availability

The datasets presented in the current study are available from the corresponding author on reasonable request.
